# Supplementary material for: Remarkable Stability Improvement of ZnO TFT with Al2O3 Gate Insulator by Yttrium Passivation with Spray Pyrolysis
Source: Nanomaterials (Basel). 2020 May 19;10(5):976. doi: 10.3390/nano10050976 (PMC7712807; doi:10.3390/nano10050976)
Supplement: Supplementary file 1 [file nanomaterials-10-00976-s001.pdf]

Supporting information

Article

Remarkable Stability Improvement of ZnO TFT with Al_2O_3 Gate Insulator by Yttrium Passivation with Spray Pyrolysis

Jewel Kumer Saha, [1,2] **Ravindra Naik Bukke,** [1] **Narendra Naik Mude,** [1] **and Jin Jang** [1*]

[1] Advanced Display Research Center (ADRC), Department of Information Display, Kyung Hee University, 26, Kyungheedae-ro, Dongdaemun-gu, Seoul 02447, Korea,

[2] Department of Physics, Jagannath University, Dhaka-1100, Bangladesh

* E-mail: jjang@khu.ac.kr

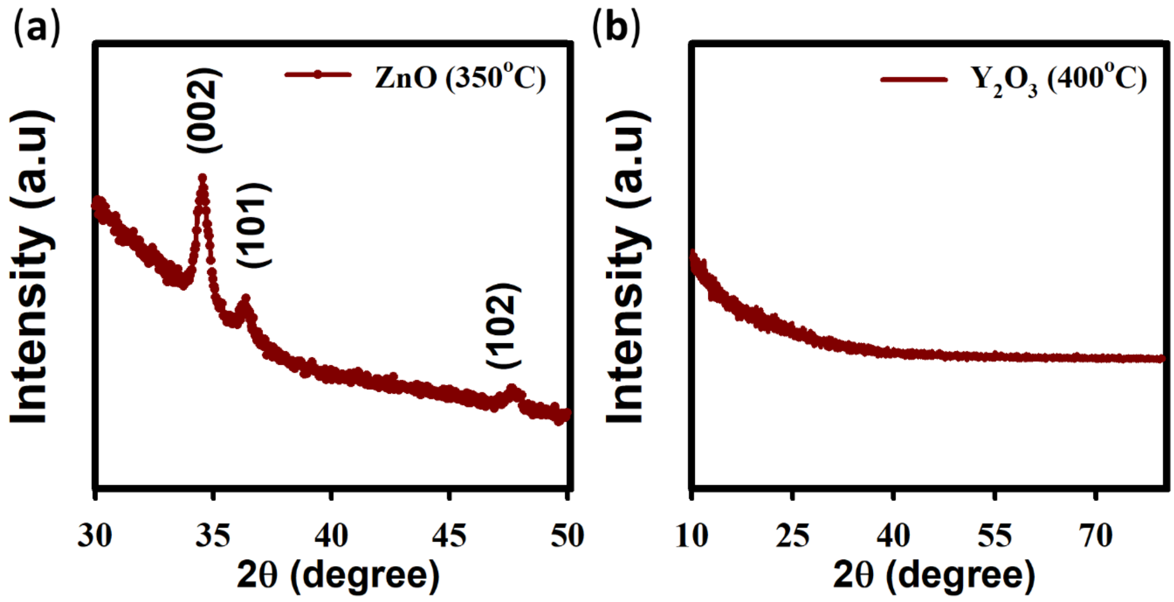

Figure S1. XRD spectra of (a) ZnO film deposited at 350°C (b) Y_2O_3 film deposited at 400°C. The three peaks corresponded to the (002), (101), and (102) planes of hexagonal wurtzite structure where the most preferred orientation is (002) plane. The Y_2O_3 film shows no prominent XRD peak confirms the amorphous nature.

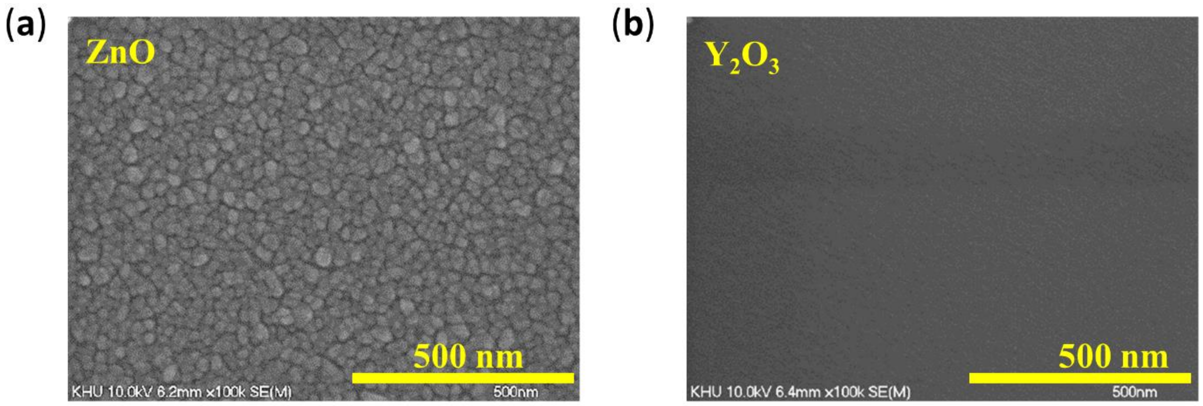

Figure S2. SEM image of (a) nano-crystalline ZnO film (b) amorphous Y_2O_3 film. The ZnO film shows compact grains with average grain size of ~30nm, which are beneficial to the transportation of electrons. The Y_2O_3 film shows no significant grains.

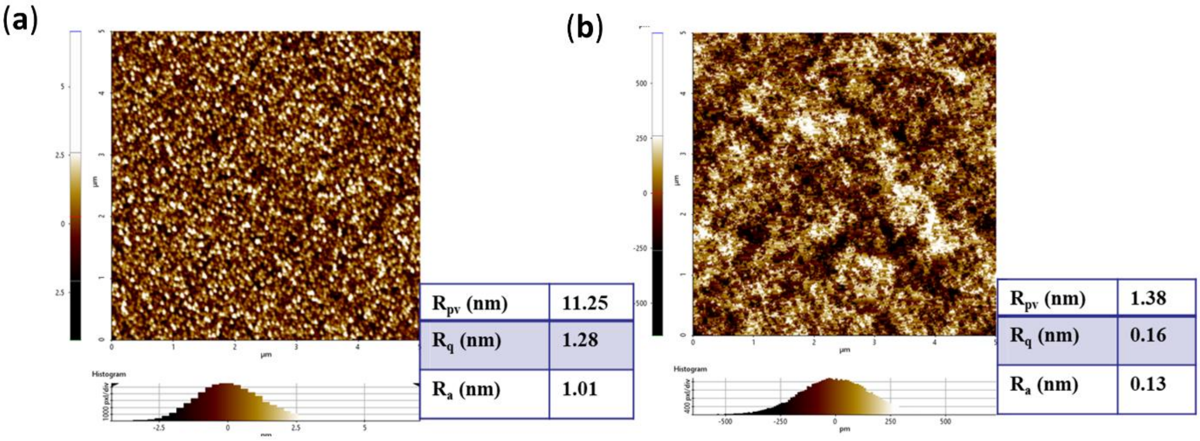

Figure S3. AFM image for surface roughness of spray coated (a) nano-crystalline ZnO film, and (b) amorphous Al_2O_3 film. Due to crystallinity, the root mean square roughness (R_{rms}) value of the ZnO film is 1.28 nm, whereas Al_2O_3 film with amorphous structure showing R_{rms} value of 0.16 nm.

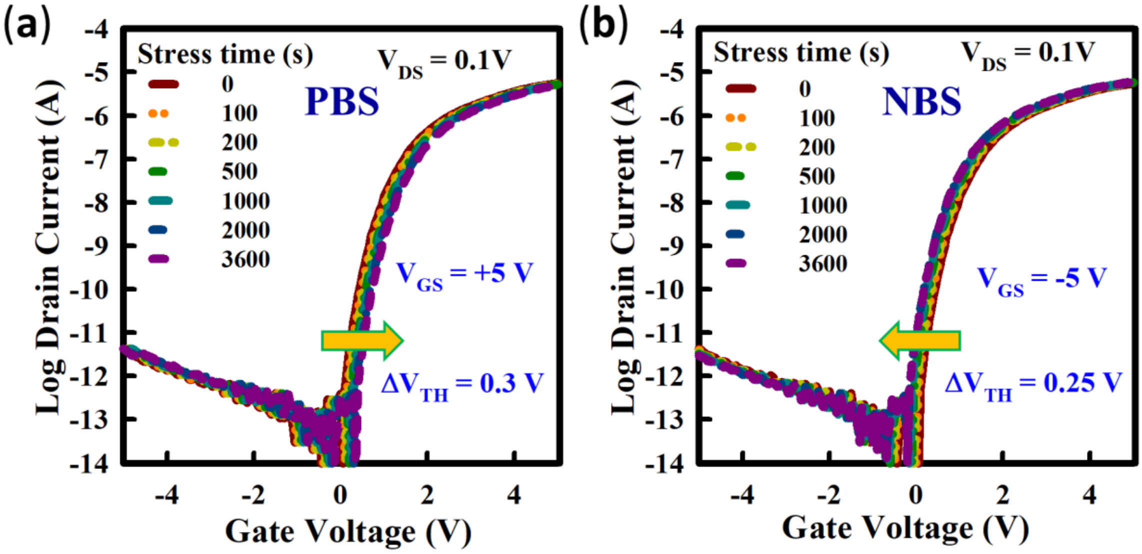

Figure S4: The evolution of transfer curve under bias stress for ZnO TFT without Y_2O_3 passivation. Transfer curves of ZnO TFTs under (a) PBS, and (b) NBS, measured by sweeping V_{GS} from -5 to +5 V at the drain voltage, V_{DS} = 0.1 V from 0 to 1 h.

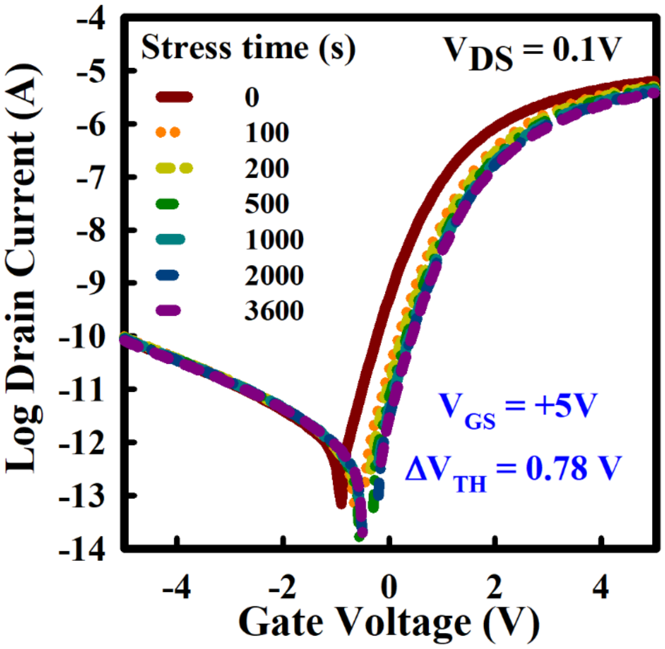

Figure S5. The evolution of transfer curve under positive bias temperature stress for ZnO TFT without Y_2O_3 passivation. The transfer curve of the TFT measured by sweeping V_{GS} from -5 to +5 V at the drain voltage, V_{DS} = 0.1 V for 1 h.The temperature stress was applied at T = 60°C.

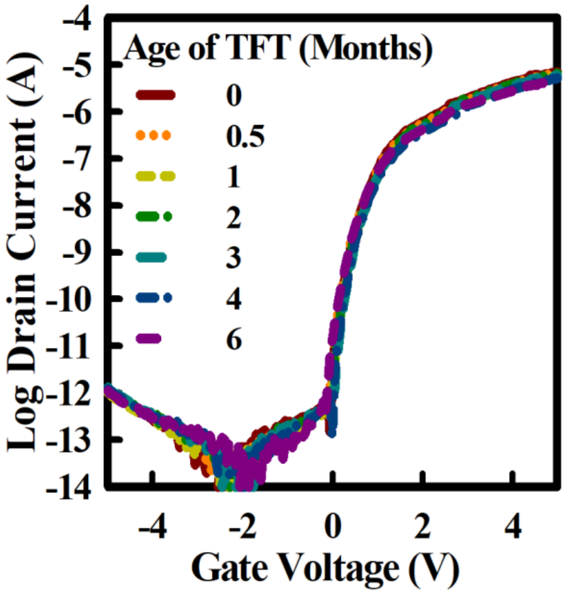

Figure S6: The evolution of transfer curve of Y_2O_3 passivated ZnO TFT in ambient air for long term stability The transfer curve of the TFT measured by sweeping V_{GS} from -5 to +5 V at the drain voltage, V_{DS} = 0.1V.
